# Complete Genome of *Serratia* sp. Strain FGI 94, a Strain Associated with Leaf-Cutter Ant Fungus Gardens

**DOI:** 10.1128/genomeA.00239-12

**Published:** 2013-03-14

**Authors:** Frank O. Aylward, Daniel M. Tremmel, Gabriel J. Starrett, David C. Bruce, Patrick Chain, Amy Chen, Karen W. Davenport, Chris Detter, Cliff S. Han, James Han, Marcel Huntemann, Natalia N. Ivanova, Nikos C. Kyrpides, Victor Markowitz, Kostas Mavrommatis, Matt Nolan, Ioanna Pagani, Amrita Pati, Sam Pitluck, Hazuki Teshima, Shweta Deshpande, Lynne Goodwin, Tanja Woyke, Cameron R. Currie

**Affiliations:** a Department of Bacteriology, University of Wisconsin-Madison, Madison, Wisconsin, USA; b Department of Energy, Great Lakes Bioenergy Research Center, University of Wisconsin-Madison, Madison, Wisconsin, USA; c Los Alamos National Laboratory, Biosciences Division, Genome Science, Los Alamos, New Mexico, USA; d DOE Joint Genome Institute, Walnut Creek, California, USA

## Abstract

*Serratia* sp. strain FGI 94 was isolated from a fungus garden of the leaf-cutter ant *Atta colombica*. Analysis of its 4.86-Mbp chromosome will help advance our knowledge of symbiotic interactions and plant biomass degradation in this ancient ant-fungus mutualism.

## GENOME ANNOUNCEMENT

The genus *Serratia* is a subgroup within the *Enterobacteriaceae* comprising isolates known to inhabit a variety of aquatic, terrestrial, and host-associated habitats (1). *Serratia* species are commonly associated with animals and plants, and some strains have been shown to cause nosocomial infections in humans (1, 2). Members of this genus are prevalent symbionts of insects, and both beneficial (3) and pathogenic (4) associations have been described.

*Serratia* sp. strain FGI 94 was isolated in 2009 from a fungus garden of the leaf-cutter ant *Atta colombica* near Pipeline Road, Panama. Leaf-cutter ants are dominant herbivores in the Neotropics that use the fresh foliar biomass they harvest to culture specialized fungus-bacterium gardens for food (5). Although these gardens are composed primarily of the obligate fungal symbiont *Leucoagaricus gongylophorus* (6), metagenomic studies have identified numerous lineages of *Enterobacteriaceae* and *Pseudomonas* that also inhabit fungus gardens (7–9). Moreover, nitrogen fixation has been shown to take place in these gardens, and bacteria of the genera *Klebsiella* and *Pantoea* have been implicated in using this process to enrich the carbon-rich forage of the ants with nitrogenous compounds (10).

The complete genome of *Serratia* sp. strain FGI 94 was sequenced at the Department of Energy (DOE) Joint Genome Institute (JGI) using Illumina technology (11). Details of library construction, sequencing, and assembly can be found on the JGI website (http://jgi.doe.gov/). Two paired-end libraries, with average insert sizes of 230 bp (17,979,030 reads, standard paired-end) and 7,839 bp (19,386,708 reads, Cre-LoxP inverse PCR [CLIP] paired-end [12]), were constructed using an Illumina HiSeq 2000, and initial assemblies were generated using a combination of Velvet v1.1.05 (13) and Allpaths, version r38445 (14). Consensus sequences were then computationally shredded into fake reads and integrated with a subset of the CLIP paired-end reads using parallel Phrap v4.24 (High Performance Software, LLC). Possible misassemblies were corrected with manual editing in Consed (15–17). Gap closure was accomplished using repeat resolution software (W. Gu, unpublished data). Additionally, 21 PCR PacBio consensus sequences (18) were completed to close gaps and to raise the quality of the final sequence. The genome of this bacterium comprises a single circular chromosome of 4.86 Mbp with an average of 954-fold coverage and a G+C content of 58.9%.

Annotation of the finished chromosome was performed using the Integrated Microbial Genomes Expert Review (IMG-ER) pipeline (19). A total of 7 copies of the 16S rRNA gene, 83 tRNAs, and 4,434 protein-coding genes were identified in this way. Comparison of the 16S rRNA genes with the NCBI 16S RNA database using BLASTn (20) revealed the invasive pathogen *Serratia rubidaea* strain JCM1240 to have the highest nucleic acid identity (99%), with the next highest matches belonging to *Serratia marcescens* subsp. *sakuensis* (98%) and *Serratia nematodiphila* strain DZ0503SBS1 (98%).

### Nucleotide sequence accession number.

The complete genome sequence of *Serratia* sp. strain FGI 94 has been deposited at DDBJ/EMBL/GenBank under the accession no. CP003942.
